# A phase 1 study in healthy volunteers to investigate the safety, tolerability, and pharmacokinetics of VIR-2482: a monoclonal antibody for the prevention of severe influenza A illness

**DOI:** 10.1128/aac.01273-23

**Published:** 2024-02-20

**Authors:** David Plotnik, Jennifer E. Sager, Madhukar Aryal, Marie C. Fanget, Alessia Peter, Michael A. Schmid, Deborah Cebrik, Erik Mogalian, Keith Boundy, Wendy W. Yeh, Paul Griffin, Maribel Reyes

**Affiliations:** 1 Vir Biotechnology, San Francisco, California, USA; 2 Humabs BioMed, SA, Vir Biotechnology, Bellinzona, Switzerland; 3 Mater Health and University of Queensland, Queensland, Australia; Providence Portland Med Ctr, Portland, Oregon, USA

**Keywords:** VIR-2482, influenza, monoclonal antibodies, prophylaxis, human immunoglobulin G1

## Abstract

The objective of this study was to evaluate the safety, tolerability, pharmacokinetics (PK), and immunogenicity of VIR-2482 in healthy adult subjects. A phase 1, first-in-human, randomized, double-blind, placebo-controlled dose-escalation study was conducted. One hundred participants were allocated to four cohorts (60 mg, 300 mg, 1,200 mg, and 1,800 mg). In each cohort, participants were randomized in a 4:1 ratio (active:placebo) to receive either VIR-2482 or volume-matched placebo by gluteal intramuscular injection. Participants remained at the investigative site under observation for 48 h, and adverse events (AEs) were collected for 56 days. PK and immunogenicity were measured up to 52 weeks post-dose. VIR-2482 was well tolerated at all doses studied. The overall incidence of AEs was comparable between VIR-2482 (68.8%) and placebo (85.0%). Nineteen VIR-2482 (23.8%) and six placebo (30.0%) recipients had Grade 1 or 2 AEs that were considered to be related to the study intervention. There were no treatment-related serious AEs. Injection-site reactions (ISRs) were reported in six (7.5%) VIR-2482 recipients, while no such reactions were reported among the placebo recipients. All ISRs were Grade 1, and there was no relationship with the dose. Median VIR-2482 serum elimination half-life ranged from 56.7 to 70.6 days across cohorts. The serum area under the curve and *C*_max_ were dose-proportional. Nasopharyngeal VIR-2482 concentrations were approximately 2%–5% of serum levels and were less than dose-proportional. The incidence of immunogenicity across all cohorts was 1.3%. Overall, the safety, tolerability, and pharmacokinetic profile of VIR-2482 at doses up to 1,800 mg supported its further investigation as a long-acting antibody for the prevention of influenza A illness. This study has been registered at ClinicalTrials.gov under identifier NCT04033406.

## INTRODUCTION

Influenza viruses are single-stranded, negative-sense, segmented RNA viruses in the *Orthomyxoviridae* family, which cause highly contagious respiratory illness (1). Influenza epidemics occur annually and result in substantial morbidity and mortality. According to the World Health Organization, each year seasonal influenza causes an estimated 3 to 5 million cases of severe disease requiring hospitalization and 290,000 to 650,000 deaths worldwide (2). Additionally, pandemic influenza strains arise unpredictably through genetic recombination in animals and have the potential to cause catastrophic public health emergencies, such as the 1918 influenza pandemic, which was estimated to have killed up to 50 million people. Influenza circulating within the human population is divided into Group A and B viruses, with Influenza A Virus (IAV) responsible for 70%–90% of seasonal infections and all known influenza pandemics (1). The majority of people infected with seasonal IAV recover without medical care; however, certain populations are at heightened risk of serious disease and death, including children under the age of 6 months, adults over the age of 65 years, and individuals of any age with comorbidities including compromised immunity, chronic obstructive pulmonary disease, asthma, or kidney disease (2). Unlike seasonal influenza, pandemic strains have historically caused high rates of serious illness and death in young and healthy populations.

The cornerstone of influenza prevention is strain-specific seasonal vaccination using inactivated viruses. Because influenza is subject to continuous antigenic drift, vaccines must be updated annually to match circulating viruses based on global surveillance data. Vaccine efficacy is difficult to predict and ranges widely from 10% to 60%, partially due to mismatches between vaccine and circulating viruses (3–5). In populations most susceptible to severe influenza disease, vaccine responses are suboptimal, and in some years, the vaccine offers no significant protection (6, 7). Additionally, certain populations cannot be vaccinated due to contraindications or forgo vaccination for personal or religious reasons (8, 9). Apart from vaccines, small-molecule antiviral drugs, including oseltamivir, zanamivir, and baloxavir marboxil, have demonstrated efficacy for post-exposure prophylaxis of influenza if started soon after exposure to an index case. However, they are not appropriate for routine seasonal influenza prevention due to the potential for drug–drug interactions, short half-life, and the emergence of drug-resistant virus strains.

Anti-influenza monoclonal antibodies (mAbs) are an emerging class of therapeutics with the potential to overcome the limitations of vaccines and antivirals. Multiple influenza proteins are possible targets neutralizing mAbs, including hemagglutinin (HA), neuraminidase (NA), and matrix protein 2. Among these, anti-HA antibodies have been extensively studied for their ability to block early phases of the virus lifecycle. HA protein consists of two functional domains: the globular head, which mediates virus attachment to host cell sialic acids, and the stem, which mediates HA maturation and endosomal fusion. Vaccine-elicited antibodies primarily target the immunodominant HA head and block virus attachment to cells; however, due to frequent mutations in the head domain, these antibody responses are type-specific and not durable. In contrast, anti-HA stem antibodies bind highly conserved structures and inhibit later steps in the virus lifecycle. HA stem antibodies are broadly neutralizing and less susceptible to viral escape but are relatively rare and difficult to elicit by vaccination. Recombinantly produced anti-HA stem mAbs are promising for influenza prophylaxis because they are likely to retain efficacy against seasonal strains without annual updates, are likely to have activity against emerging pandemic strains, and may be administered by passive immunization to protect patients with poor immune responses to vaccines.

Multiple anti-HA stem mAbs have been discovered and advanced to clinical trials, including VIS410, MHAA4549A, MHAB5553A, CR6261, and MEDI8852 [reviewed in reference (10)]. All these have been developed for influenza treatment when administered by intravenous infusion within several days of infection, and three have been evaluated in phase 2 trials; however, none demonstrated significant clinical benefit for influenza treatment at doses up to 3,000 mg (MEDI8852), 4,000 mg (VIS410), or 8,400 mg (MHAA4549A) (11–13). The lack of efficacy observed in these studies may be due to drug administration occurring too late after the onset of infection, as these trials allowed enrollment 3–5 days after symptom onset with actual infection likely occurring days prior to symptoms. As the clinical syndrome of active influenza is driven more by host immune response than viral replication later in the disease process (14), mAbs against influenza may be unlikely to demonstrate efficacy when administered well after the onset of symptoms. This hypothesis is consistent with the demonstrated clinical efficacy of VIS410 when administered 24 h after viral inoculation in a human challenge study (15) while failing in the aforementioned phase 2 trial. Also consistent with this premise, mAbs targeting severe acute respiratory syndrome coronavirus 2 (SARS-CoV-2) demonstrated robust clinical efficacy early in the disease process (16, 17) but not later in hospitalized patients (18). Thus, it is plausible that anti-influenza antibodies may be effective when administered early in the disease process or for prophylaxis if sufficient drug concentrations are present prior to viral exposure. Notably, passive immunization with mAbs has been successfully used for the prevention of other respiratory pathogens, including palivizumab and nirsevimab for respiratory syncytial virus and tixagevimab/cilgavimab for SARS-CoV-2.

VIR-2482 is a mAb that was engineered for influenza prophylaxis. VIR-2482 was derived from MEDI8852, which targets a highly conserved HA stem epitope and has broad reactivity to all 18 known HA proteins (19). VIR-2482 was generated by introducing the ”LS mutation” (M428L/N434S) into the Fc region of MEDI8852 to extend elimination half-life through increased neonatal Fc receptor (FcRn)-mediated antibody recirculation. These enhanced pharmacokinetic (PK) properties potentially enable VIR-2482 to be dosed once per influenza season by intramuscular (IM) injection in an outpatient setting, in contrast to other anti-HA mAbs that are administered intravenously. Passive immunization with VIR-2482 represents a novel strategy for the prevention of seasonal influenza, especially in populations afforded inadequate protection by vaccines or those unable to take vaccines, and represents a medical countermeasure that could be rapidly deployed in response to pandemic influenza. Here, we report the results of a first-in-human phase 1 clinical trial conducted to assess the safety, tolerability, PK, and immunogenicity of VIR-2482 in healthy adults.

### Role of the funding source

The sponsor, Vir Biotechnology Inc., oversaw the study design and analyzed data; the investigators and sponsor jointly conducted the trial and collected data. The authors and sponsor jointly decided to submit the manuscript for publication. Authors had access to relevant aggregated study data and other information (including the study protocol, analysis plans, validated data tables, and clinical study report) required to understand and report research findings. The authors take responsibility for the presentation and publication of the research findings, have been fully involved at all stages of publication and presentation development, and are willing to take public responsibility for all aspects of the work. All individuals included as authors and contributors who made substantial intellectual contributions to the research, data analysis, and publication or presentation development are listed appropriately. The role of the sponsors in the design, execution, analysis, reporting, and funding is fully disclosed. The authors’ personal interests, financial or non-financial, relating to this research and its publication have been disclosed.

## MATERIALS AND METHODS

### Trial overview

This trial was a phase 1, first-in-human, randomized, double-blind, placebo-controlled dose-escalation study designed to determine the safety, tolerability, PK, and immunogenicity of VIR-2482 vs placebo in healthy adults. The study was conducted in accordance with consensus ethical principles derived from international guidelines, including the Declaration of Helsinki and Council for International Organizations of Medical Sciences, applicable International Council for Harmonization, and Good Clinical Practice and applicable laws and regulations. Informed consent was obtained from all participants prior to their participation in the study. The study is reported using Consolidated Standards of Reporting Trials (CONSORT) guidelines.

### Participants

The study was conducted at one study center in Australia and enrolled 101 healthy male and female volunteers between 18 and 65 years of age without acute or chronic medical conditions and with a body mass index (BMI) of 18–32 kg/m^2^, inclusive. Exclusion criteria included receipt of any immunoglobulin within 6 months prior to randomization and receipt of any mAb. Full inclusion and exclusion criteria are presented in Appendix A—Study Inclusion and Exclusion Criteria.

### Trial design

**Fig 1:**
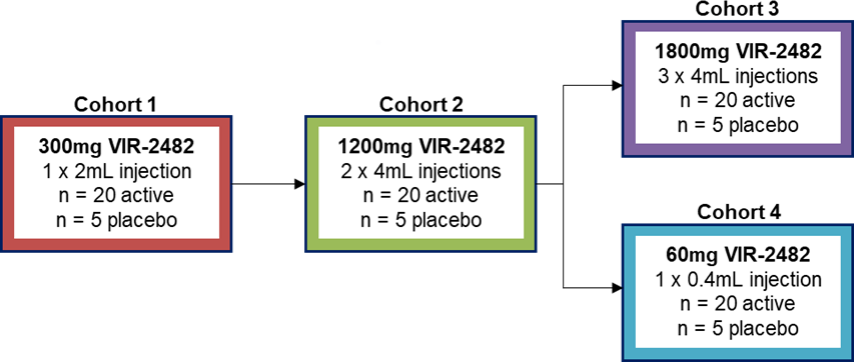
Study schema.

Participants were allocated to one of the four dosing cohorts: 300 mg (Cohort 1), 1,200 mg (Cohort 2), 1,800 mg (Cohort 3), and 60 mg (Cohort 4). Within each cohort, participants were randomized in a 4:1 (active:placebo) ratio. Cohorts were dosed sequentially (Fig. 1). Participants, investigators, and all site personnel were blinded to study intervention until the end of the study. Designated personnel onsite who were not directly involved with participant care were aware of randomization codes and prepared and dispensed study intervention.

**TABLE 1:** Baseline demographics^*a*^

Characteristics	VIR-2482 60 mg (*N* = 20)	VIR-2482 300 mg (*N* = 20)	VIR-2482 1,200 mg (*N* = 20)	VIR-2482 1,800 mg (*N* = 20)	VIR-2482 overall (*N* = 80)	Pooled placebo (*N* = 20)	Total (*N* = 100)
Sex [*n* (%)]							
Female	6 (30.0)	9 (45.0)	10 (50.0)	8 (40.0)	33 (41.3)	11 (55.0)	44 (44.0)
Male	14 (70.0)	11 (55.0)	10 (50.0)	12 (60.0)	47 (58.8)	9 (45.0)	56 (56.0)
Age, y [mean (SD)]	37.2 (14.5)	29.3 (11.6)	28.9 (10.7)	29.9 (11.4)	31.3 (12.4)	30.7 (12.8)	31.2 (12.4)
Race [*n* (%)]							
American Indian or Alaska Native	0	0	0	0	0	0	0
Asian	2 (10.0)	1 (5.0)	2 (10.0)	4 (20.0)	9 (11.3)	1 (5.0)	10 (10.0)
Black or African American	0	1 (5.0)	0	0	1 (1.3)	0	1 (1.0)
Native Hawaiian or other Pacific Islander	0	0	0	2 (10.0)	2 (2.5)	0	2 (2.0)
White	17 (85.0)	15 (75.0)	13 (65.0)	12 (60.0)	57 (71.3)	17 (85.0)	74 (74.0)
Multiple	0	1 (5.0)	0	0	1 (1.3)	1 (5.0)	2 (2.0)
Other	1 (5.0)	2 (10.0)	5 (25.0)	2 (10.0)	10 (12.5)	1 (5.0)	11 (11.0)
Ethnicity [*n* (%)]							
Hispanic or Latino	2 (10.0)	3 (15.0)	5 (25.0)	0	10 (12.5)	1 (5.0)	11 (11.0)
Not Hispanic or Latino	18 (90.0)	14 (70.0)	12 (60.0)	17 (85.0)	61 (76.3)	18 (90.0)	79 (79.0)
Not reported	0	3 (15.0)	3 (15.0)	3 (15.0)	9 (11.3)	1 (5.0)	10 (10.0)
BMI, kg/m^2^ [mean (SD)]	26.1 (3.1)	24.7 (3.2)	24.3 (2.6)	25.2 (3.1)	25.1 (3.0)	24.1 (3.7)	24.9 (3.2)

^*a*^ n, number; SD, standard deviation; y, years.

### Product description

VIR-2482 is an engineered human IgG1k antibody expressed in Chinese Hamster Ovary cells, formulated as a lyophilized solid. The formulation was reconstituted with sterile water for injection to 150 mg/mL with a pH of 6.0.

### Study intervention

Participants were administered VIR-2482 or volume-matched placebo as an IM injection in dorsogluteal or ventrogluteal sites. Participants in Cohort 1 (300 mg) received a single 2 mL injection, participants in Cohort 2 (1,200 mg) received two injections of 4 mL each, participants in Cohort 3 (1,800 mg) received three injections of 4 mL each, and participants in Cohort 4 (60 mg) received a single 0.4 mL injection. A maximum of 4 mL volume was used per injection site. The first two participants in each cohort were randomized 1:1 to receive either VIR-2482 or placebo and were monitored for 24 h prior to dosing the remaining participants in the cohort.

### Study endpoints

The primary endpoint was the safety and tolerability of VIR-2482 after a single dose, as assessed by the incidence of treatment-emergent adverse events (TEAEs) and clinical assessments. Secondary endpoints included characterization of serum PK and immunogenicity response after a single dose of VIR-2482. Characterization of nasopharyngeal PK was included as an exploratory endpoint.

#### Safety assessments

All adverse events (AEs) were collected for 56 days post-dose, and serious AEs were collected for the duration of study participation. Full physical examinations were conducted during screening and the last scheduled visit (week 24) and included head, neck, respiratory, cardiovascular, gastrointestinal, extremity, skin, and neurological assessments. Physical examinations were followed by collecting vital signs, including blood pressure, pulse rate, temperature, respiratory rate, and oxygen saturation. On day 1, 12-lead electrocardiograms (ECG) were performed <15 min pre-dose and <30 min post-dose. Clinical laboratory assessments included routine hematology, serum chemistry, liver function tests, and urinalysis performed by the local laboratory associated with the clinical site. Abnormal values for selected lab parameters were programmatically graded using criteria based on the Common Terminology for Adverse Events (CTCAE), Version 5.0. Local injection site tolerability was assessed at 30 min, 2 h, 12 h, 24 h, and 48 h post-injection. A review of cumulative blinded safety data was performed prior to dose escalation to the next cohort.

#### Pharmacokinetic assessments

Blood was collected to assess serum concentrations of VIR-2482 prior to dosing, at hours 1, 2, 6, 12, 24, and 48, and at weeks 1, 2, 4, 6, 8, 12, 20, 34, and 52 post-dose. VIR-2482 concentrations in serum were determined using a validated electrochemiluminescence method, which used anti-idiotypic antibodies specific to VIR-2482 for capture and detection. The quantification range of this assay was 0.050–50.0 µg/mL.

Nasopharyngeal swabs (NPS) were collected prior to dosing, at hours 2, 12, 24, and 48, and at weeks 1, 2, 4, 8, 12, and 34 post-dose. Each swab was mixed with a 3 mL universal transport medium (Copan Diagnostics) and aliquoted. One aliquot was used for the measurement of VIR-2482 similar to the serum PK assay described above. Another aliquot was used to measure urea concentration via a chromogenic assay (EIABUN, Thermo Fisher). Urea was also measured in a serum sample collected at the same time as the NPS sample. Quantification ranges of urea in NPS and serum were 0.039–5.0 mg/dL and 0.313–10.0 mg/dL, respectively. Dilution factors were calculated as the ratio between serum urea and NPS urea concentrations. VIR-2482 NPS concentrations were normalized by multiplying measured nasal VIR-2482 concentration by the dilution factors. The quantification range of the NPS PK assay was 0.009–4.444 µg/mL.

Non-compartmental methods were used to estimate PK parameters from both serum and NPS PK concentration data. Analyses were performed using Phoenix WinNonlin version 8.3.5 (Certara USA, Inc., Princeton, NJ). Actual sampling times were used, and data below the limit of quantification (BLQ) were excluded from analysis, except for NPS PK data that were additionally analyzed using the M5 and M7 data imputation rules (20). Serum PK parameters included maximum serum concentration (*C*_max_), time to reach maximum concentration (*T*_max_), predicted serum concentration 180 days post-dose (*C*_180_), the area under the concentration–time curve from 0 to 180 days (AUC_0–180_), the area under the concentration–time curve extrapolated to infinity (AUC_INF_), terminal elimination half-life (*t*_1/2_), apparent total-body clearance (CL/*F*), and apparent volume of distribution (*V*_*z*_/*F*). AUC was computed using the “linear-up, log-down” trapezoidal rule, and AUC_INF_ values were excluded when >20% of the AUC was extrapolated. NPS PK parameters were calculated in the same manner as serum PK parameters and included *C*_max_, *C*_180_, *T*_max_, AUC_0–180_, and *t*_1/2_.

#### Immunogenicity assessments

Serum was collected pre-dose and at weeks 2, 4, 8, 20, 34, and 52 post-dose. A three-tier approach (screen, confirm, and titer) was used to detect anti-drug antibodies (ADA) to VIR-2482. Validated electrochemiluminescent bridging assays were used to analyze the samples. The assay cut point was established using serum from drug-naive healthy individuals (*n* = 50) with a screening cut point factor of −1.102. The screening assay had a sensitivity of 2.37 ng/mL using anti-VIR-2482 antibody-positive control. The assay drug tolerance was 25 µg/mL of VIR-2482 at 50 ng/mL of positive control and 200 µg/mL of VIR-2482 at 2,000 ng/mL of positive control.

### Statistical analyses

This study was not powered for inferential statistical analysis, and no hypothesis testing was planned or performed. Data collected were anticipated to provide sufficient observation of safety and PK to expand later into a larger population to establish proof-of-concept. Safety and tolerability data for participants receiving placebo were combined across cohorts to form a pooled placebo group. AEs were coded using the Medical Dictionary for Regulatory Activities, Version 24.1, and graded using the CTCAE, Version 5.0.

## RESULTS

**Fig 2:**
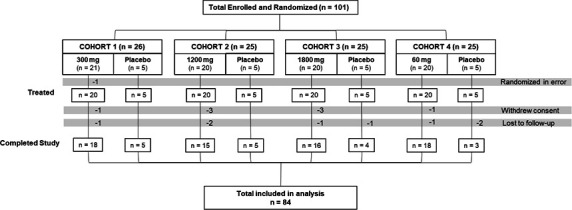
CONSORT diagram of participant disposition in the VIR-2482-3001 study.

Between August 2019 and October 2020, a total of 101 healthy adults were enrolled in the study. Of these, one was randomized in error and was withdrawn prior to receiving study intervention. Following dosing, eight participants were lost to follow-up and eight withdrew consent. Overall, 83.2% of participants completed the study and completion rates were balanced between groups. A total of 80 participants received VIR-2482 and 20 participants received placebo (Fig. 2).

Participant demographics were balanced across cohorts. Participants were 56.0% male and identified predominantly as White (74.0%) and not of Hispanic or Latino ethnicity (79.0%). Participants had a median age of 28 years and age ranged from 18 to 63 years, with no participant older than 65 years of age. The median BMI was 25.2 kg/m^2^ and was balanced across cohorts with BMI ranging from 18.0 to 31.8 kg/m^2^. Full participant demographics are presented in Table 1.

### Safety and tolerability

VIR-2482 administered by IM injection was generally safe and well tolerated at doses up to 1,800 mg (Table 2). The overall proportion of AEs was comparable between participants who received VIR-2482 (68%) and placebo (85%). Most AEs were Grade 1 or 2 in severity, with no dose-dependent trends observed. No AEs led to study discontinuation. Nineteen (23.8%) VIR-2482 recipients and six (30%) placebo recipients had AEs considered related to study intervention by the investigator. The most common treatment-related TEAEs in VIR-2482 cohorts were headache (7.5%; 6 of 80 participants), cough (5.0%; 4 of 80 participants), and presyncope, viral upper respiratory tract infection, and oropharyngeal pain (each 2.5%; 2 of 80 participants), with all other TEAEs occurring in no more than one participant. All treatment-related TEAEs were Grade 1 or 2 in severity and resolved without sequelae. No treatment-related laboratory or ECG abnormalities were observed.

**TABLE 2:** Summary of VIR-2482 safety^*a*^

TEAE category	VIR-2482 60 mg (*N* = 20)	VIR-2482 300 mg (*N* = 20)	VIR-2482 1,200 mg (*N* = 20)	VIR-2482 1,800 mg (*N* = 20)	VIR-2482 overall (*N* = 80)	Pooled placebo (*N* = 20)	Total (*N* = 100)
	*n* (%)	*n* (%)	*n* (%)	*n* (%)	*n* (%)	*n* (%)	*n* (%)
Any AE	15 (75.0)	12 (60.0)	14 (70.0)	14 (70.0)	55 (68.8)	17 (85.0)	72 (72.0)
Maximum CTCAE toxicity grade							
Grade 1 mild	12 (60.0)	9 (45.0)	9 (45.0)	12 (60.0)	42 (52.5)	14 (70.0)	56 (56.0)
Grade 2 moderate	2 (10.0)	3 (15.0)	4 (20.0)	2 (10.0)	11 (13.8)	3 (15.0)	14 (14.0)
Grade 3 severe	0	0	1 (5.0)	0	1 (1.3)	0	1 (1.0)
Grade 4 life-threatening	1 (5.0)	0	0	0	1 (1.3)	0	1 (1.0)
Grade 5 death	0	0	0	0	0	0	0
Any AE with the outcome of death	0	0	0	0	0	0	0
Any SAE	1 (5.0)	0	0	0	1 (1.3)	0	1 (1.0)
Any TEAEs	4 (20.0)	5 (25.0)	5 (25.0)	5 (25.0)	19 (23.8)	6 (30.0)	25 (25.0)
Any AE leading to study discontinuation	0	0	0	0	0	0	0
Any AE leading to premature discontinuation from the study	0	0	0	0	0	0	0

^*a*^ For each category, participants are included only once, even if they experienced multiple events in that category. AE, adverse event; CTCAE, common terminology for AE; *n*, number; SAE, serious AE; TEAE, treatment-emergent AE.

Two Grade 3 (severe) non-serious AEs of syncope and skin laceration occurred in one participant 42 days after dosing with 1,200 mg VIR-2482. A Grade 4 (life-threatening) serious AE of coronary artery atherosclerosis occurred in one participant 298 days after dosing with 60 mg VIR-2482; the participant underwent a coronary artery bypass procedure, and the event was considered resolved. No Grade 3 or 4 events were considered treatment-related. No deaths occurred during the study.

Local injection site reactions (ISRs) were reported in 6 of 80 (7.5%) VIR-2482 recipients across all doses and included symptoms of bruising, pain/tenderness, redness, and swelling. All ISRs were Grade 1 (mild), with no apparent relationship between ISR incidence and dose, and all ISRs resolved spontaneously. No participants receiving placebo had an ISR.

### Pharmacokinetics

**Fig 3:**
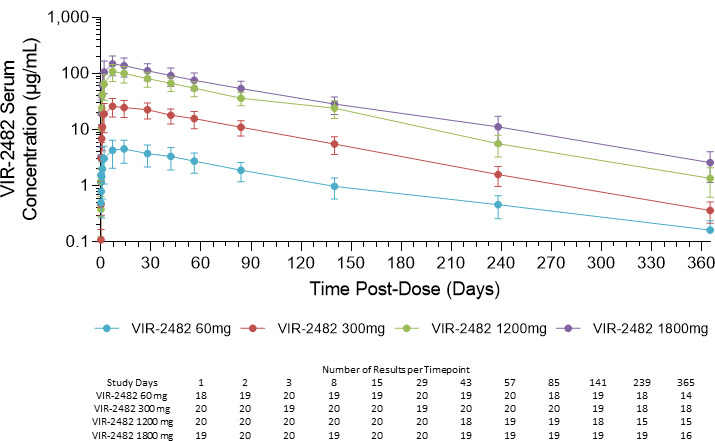
VIR-2482 serum pharmacokinetics. Symbols indicate the mean and standard deviation VIR-2482 serum concentration at each time point. The number of results included at each time point is presented in the table below the figure.

Serum PK data are presented in Fig. 3 and Table 3. Median *T*_max_ following IM injection was ~7 days for doses of 300 mg, 1,200 mg, and 1,800 mg, and 12.5 days for 60 mg. The geometric mean volume of distribution (*V*_*z*_/*F*) was 10.6–15.1 L across dosing cohorts. Median VIR-2482 serum elimination half-life was 56.7–70.6 days across dosing cohorts. VIR-2482 serum *C*_max_ and AUC_INF_ were dose-proportional across the range of 60 mg to 1,800 mg.

**TABLE 3:** Serum pharmacokinetic parameters^*a*^

PK parameter	VIR-2482 60 mg (*N* = 20)	VIR-2482 300 mg (*N* = 20)	VIR-2482 1,200 mg (*N* = 20)	VIR-2482 1,800 mg (*N* = 20)
*C*_max_ (μg/mL)				
*n*	20	20	20	20
Geometric mean (geometric CV%)	3.9 (74.7)	24.9 (40.0)	104 (35.7)	139 (48.3)
*C*_180_ (μg/mL)				
*n*	20	20	19	19
Geometric mean (geometric CV%)	0.7 (48.0)	3.2 (42.7)	13.1 (37.2)	17.8 (46.6)
*T*_max_ (day)				
*n*	20	20	20	20
Median (min, max)	12.5 (7.0, 42.1)	7.05 (6.1, 28.2)	7.03 (6.0, 13.1)	7.1 (2.0, 14.9)
AUC_0–180_ (day*μg/mL)				
*n*	20	20	19	19
Geometric mean (geometric CV%)	339 (59.2)	2,040 (36.8)	7,790 (31.0)	10,400 (42.0)
AUC_INF_ (day*μg/mL)				
*n*	19	20	18	19
Geometric mean (geometric CV%)	411 (54.7)	2,310 (37.4)	8,860 (32.1)	12,100 (43.4)
CL/F (mL/day)				
*n*	19	20	18	19
Geometric mean (geometric CV%)	146 (54.7)	130 (37.4)	135 (32.1)	149 (43.4)
*V*_*z*_/*F* (L)				
*n*	19	20	18	19
Geometric mean (geometric CV%)	15.1 (63.7)	10.6 (35.4)	11.1 (32.0)	13.4 (41.4)
*t*_1/2_ (day)				
*n*	20	20	19	19
Median (min, max)	70.6 (49.2, 99)	57.1 (51.2, 64.9)	56.7 (45.8, 72.8)	61.1 (51.4, 77.4)

^*a*^ *C*_max_, maximum observed serum concentration; *C*_180_, predicted serum concentration at day 180; *T*_max_, time to reach maximum concentration; AUC_0–180_, partial area under the concentration–time curve from time 0 to 180 days; AUC_INF_, area under the concentration–time curve from time 0 to time infinity; CL/F, apparent total-body clearance following extravascular dosing; *V*_*z*_/*F*, apparent volume of distribution estimated during the terminal elimination phase, following extravascular dosing; *t*_1/2_, terminal elimination half-life.

**Fig 4:**
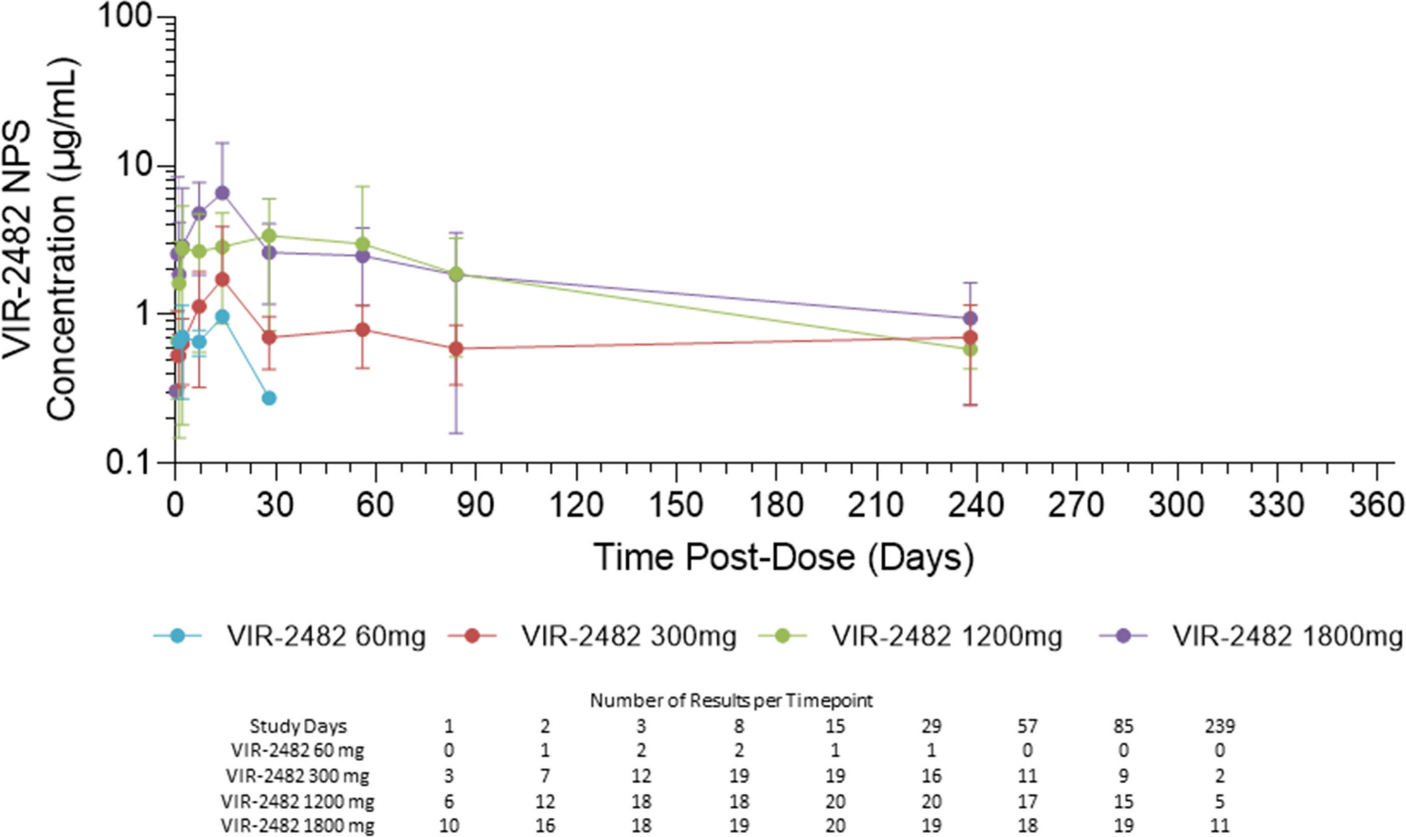
VIR-2482 nasopharyngeal pharmacokinetics. Symbols indicate the mean and standard deviation VIR-2482 NPS concentrations at each time point. The number of results included at each timepoint is presented in the table below the figure.

NPS PK data are presented in Fig. 4 and Table 4. Across all cohorts, VIR-2482 was BLQ in 88% of samples collected at 2 h and 12 h, 80% of samples collected at day 240, and 96% of samples from all timepoints in the 60 mg cohort. NPS results were analyzed by three methods: excluding BLQ data (M1), imputing ½ the lower limit of quantification (M5), or substituting zero for BLQ (M7). Results from the M1 approach are presented in Fig. 4 and Table 4, and results from approaches M5 and M7 are presented in Supplemental figures and tables. Across the 300 mg, 1,200 mg, and 1,800 mg cohorts and timepoints between days 1 and 84, VIR-2482 concentrations in the nasopharynx were approximately 2%–5% of within-subject serum levels. Median NPS *T*_max_ ranged from 7 to 20 days and median NPS elimination half-life ranged from 55 to 80.9 days across cohorts. NPS exposures were less than dose-proportional.

**TABLE 4:** Nasopharyngeal pharmacokinetic parameters^*a*^

PK parameter	VIR-2482 60 mg (*N* = 20)	VIR-2482 300 mg (*N* = 20)	VIR-2482 1,200 mg (*N* = 20)	VIR-2482 1,800 mg (*N* = 20)
*C*_max_ (μg/mL)				
*n*	5	20	20	20
Geometric mean (geometric CV%)	0.6 (56.6)	1.3 (96.8)	3.8 (106.0)	5.9 (88.5)
*C*_180_ (μg/mL)				
*n*	0	9	12	18
Geometric mean (geometric CV%)	NC	0.295 (106.3)	0.282 (627.7)	0.716 (121.8)
*T*_max_ (day)				
*n*	5	20	20	20
Median (min, max)	7.0 (1, 28.1)	13.4 (1.0, 83.1)	20 (1, 56.3)	11 (0.5, 28.2)
AUC_0–180_ (day*μg/mL)				
*n*	0	9	12	18
Geometric mean (geometric CV%)	NC	120 (40.0)	294 (66.5)	328 (70.8)
*t*_1/2_ (day)				
*n*	0	7	11	17
Median (min, max)	NC	74 (51.8, 2170)	55 (13.7, 2960)	80.9 (30.5, 2180)

^*a*^ *C*_max_, maximum observed NPS concentration; *C*_180_, predicted NPS concentration at Day 180. *T*_max_, time to reach; maximum concentration; AUC_0–180_, partial area under the concentration–time curve from time 0 to 180 days; *t*_1/2_, terminal elimination half-life. CV, coefficient of variation; n, number; NC, not calculated; NPS, nasopharyngeal secretion.

### Immunogenicity

The overall incidence of ADA was low (6/80, 8% of all participants). Of these, five participants had pre-existing (pre-dose) ADA. Transient treatment-induced ADA was detected in one participant, and in this participant ADA titer was low (1:8). ADA was not correlated with changes in PK or with safety events.

## DISCUSSION

This phase 1 study established a favorable safety, tolerability, and PK profile for VIR-2482 administered by IM injection at doses up to 1,800 mg. Safety data from healthy volunteers collected across multiple dosing cohorts for 1-year post-dose demonstrated that VIR-2482 was well tolerated following intramuscular administrations ranging from a single 0.4 mL injection to three separate 4 mL injections. Immunogenicity risk was low as ADA were infrequent, transient, and not correlated with observable impacts on PK or safety. The serum elimination half-life of VIR-2482 ranged from 56.7 to 70.6 days across cohorts, representing a threefold increase compared to the parental antibody MEDI8852 (half-life 19–21 days) (13). Serum exposures were dose-proportional, consistent with the observation VIR-2482 does not have known binding to endogenous antigens (data on file).

The relationship between serum and tissue drug concentrations is a critical factor for estimating clinically effective doses. Neutralizing concentrations of VIR-2482 in upper airway mucosa could block aerosol transmission and prevent infection, while neutralizing concentrations in lower airways would not block initial infection but may prevent symptomatic illness through inhibiting viral replication, immune activation, and lung tissue damage. In this study, the distribution of VIR-2482 to upper airway mucosa was confirmed in nasal swabs, with serum-to-nasopharynx partitioning ranging from 2% to 5%. Additionally, NPS exposures were less than dose-proportional, suggesting VIR-2482 distribution to the nasopharynx may be saturable. These mucosal biodistribution properties are consistent with reports for other mAbs (21, 22), which indicates that enhanced FcRn binding of VIR-2482 did not significantly alter antibody translocation to mucosal surfaces. Antibody distribution to lower airway fluid is believed to be higher than the nasopharynx, with partitioning estimates ranging from 5% to 31% (23–25). VIR-2482 distribution to lower airway fluid is likely to be within this range, however, additional studies would be required to confirm this.

Following positive results from this first-in-human study, VIR-2482 was advanced to a phase 2 efficacy trial (VIR-2482-4002; ClinicalTrials.gov: NCT05567783). Prevention of symptomatic influenza illness was predicted to be achieved to various degrees by targeting a serum concentration range of 8–44 µg/mL, based on the *in vitro* neutralizing activity of VIR-2482 and assumed tissue partitioning across the respiratory tract of 5%–25%. The neutralizing properties of VIR-2482 and its parental antibody MEDI8852 are the same (data on file), and MEDI8852 has been described previously (19). Doses of 450 mg and 1,200 mg were selected for further investigation in phase 2; 1,200 mg was judged to be the highest clinically feasible IM dose (two separate 4 mL injections), predicted to maintain the 8 µg/mL target for >6 months and the 44 µg/mL target for 3 months, while 450 mg was chosen to maintain the 8 µg/mL target for the majority of an active influenza season.

Populations anticipated to benefit most from antibody prophylaxis include adults over the age of 65 years and immunocompromised individuals. This phase 1 study was limited to a small population of healthy volunteers, therefore, additional studies were conducted to investigate VIR-2482 in larger and more diverse populations. In addition to phase 2, in which VIR-2482 was administered to thousands of participants between ages 18 and 65 years, a phase 1b study (VIR-2482-4004) was also conducted in adults over the age of 65. These studies assessed the impacts of race, ethnicity, sex, age, and other factors on the safety and PK of VIR-2482 and will be described separately.

While multiple HA-stem antibodies have been investigated for influenza treatment, VIR-2482 is the first to be tested for pre-exposure prophylaxis. The combined findings from this phase 1 study and subsequent VIR-2482 trials provide foundational data regarding the safety and pharmacology of antibodies for influenza prophylaxis. Notably, next-generation influenza antibodies targeting neuraminidase are currently under development (26) and will benefit from lessons learned in the development of VIR-2482. Neuraminidase inhibition is a clinically validated mechanism of action, and NA-targeting mAbs either alone or in combination with HA-targeting mAbs have great promise for influenza prophylaxis. Overall, in conjunction with existing vaccines and antivirals, long-acting mAbs against influenza have the potential to play an important role in the prevention of seasonal influenza and as medical countermeasures against pandemic influenza.

## AFTER EPUB

*[Minor changes in wording were made to the abstract on 27 February 2024.]*

## SUPPLEMENTAL MATERIAL

Fig. S1 and S2 and Tables S1 and S2 aac.01273-23-s0001.docx Nasopharyngeal PK data analyzed by alternate (M5/M7) methods.

Appendix S1 aac.01273-23-s0002.docx Study inclusion/exclusion criteria.

ASM does not own the copyrights to Supplemental Material that may be linked to, or accessed through, an article. The authors have granted ASM a non-exclusive, world-wide license to publish the Supplemental Material files. Please contact the corresponding author directly for reuse.

## References

[1] Rambaut A, Pybus OG, Nelson MI, Viboud C, Taubenberger JK, Holmes EC. 2008. The genomic and epidemiological dynamics of human influenza A virus. Nature 453:615–619. doi:10.1038/nature06945 18418375 PMC2441973

[2] World Health Organization. 2023. Influenza (seasonal). Available from: https://www.who.int/news-room/fact-sheets/detail/influenza-(seasonal). Retrieved 15 Sep 2023.

[3] Paules CI, Sullivan SG, Subbarao K, Fauci AS. 2018. Chasing seasonal influenza - the need for a universal influenza vaccine. N Engl J Med 378:7–9. doi:10.1056/NEJMp1714916 29185857

[4] Centers for Disease Control and Prevention. 2023. Vaccine effectiveness studies. Available from: https://www.cdc.gov/coronavirus/2019-ncov/vaccines/effectiveness/how-they-work.html. Retrieved 15 Sep 2023.

[5] McLean HQ, Belongia EA. 2021. Influenza vaccine effectiveness: new insights and challenges. Cold Spring Harb Perspect Med 11:a038315. doi:10.1101/cshperspect.a038315 31988202 PMC8168527

[6] Krammer F, Smith GJD, Fouchier RAM, Peiris M, Kedzierska K, Doherty PC, Palese P, Shaw ML, Treanor J, Webster RG, García-Sastre A. 2018. Influenza. Nat Rev Dis Primers 4:3. doi:10.1038/s41572-018-0002-y 29955068 PMC7097467

[7] Centers for Disease Control and Prevention. 2023. Past seasons’ vaccine effectiveness estimates. Available from: https://www.cdc.gov/flu/vaccines-work/past-seasons-estimates.html. Retrieved 15 Sep 2023.

[8] Kabir KMA, Jusup M, Tanimoto J. 2019. Behavioral incentives in a vaccination-dilemma setting with optional treatment. Phys Rev E 100:062402. doi:10.1103/PhysRevE.100.062402 31962423

[9] Gaitonde DY, Moore FC, Morgan MK. 2019. Influenza: diagnosis and treatment. Am Fam Physician 100:751–758. 31845781

[10] Sedeyn K, Saelens X. 2019. New antibody-based prevention and treatment options for influenza. Antiviral Res 170:104562. doi:10.1016/j.antiviral.2019.104562 31323236

[11] Hershberger E, Sloan S, Narayan K, Hay CA, Smith P, Engler F, Jeeninga R, Smits S, Trevejo J, Shriver Z, Oldach D. 2019. Safety and efficacy of monoclonal antibody VIS410 in adults with uncomplicated influenza A infection: results from a randomized, double-blind, phase-2, placebo-controlled study. EBioMedicine 40:574–582. doi:10.1016/j.ebiom.2018.12.051 30638863 PMC6412085

[12] Lim JJ, Dar S, Venter D, Horcajada JP, Kulkarni P, Nguyen A, McBride JM, Deng R, Galanter J, Chu T, Newton EM, Tavel JA, Peck MC. 2022. A phase 2 randomized, double-blind, placebo-controlled trial of the monoclonal antibody MHAA4549A in patients with acute uncomplicated influenza A infection. Open Forum Infect Dis 9:ofab630. doi:10.1093/ofid/ofab630 35106315 PMC8801227

[13] Ali SO, Takas T, Nyborg A, Shoemaker K, Kallewaard NL, Chiong R, Dubovsky F, Mallory RM. 2018. Evaluation of MEDI8852, an anti-influenza A monoclonal antibody, in treating acute uncomplicated influenza. Antimicrob Agents Chemother 62:e00694-18. doi:10.1128/AAC.00694-18 30150460 PMC6201130

[14] Gounder AP, Boon ACM. 2019. Influenza pathogenesis: the effect of host factors on severity of disease. J Immunol 202:341–350. doi:10.4049/jimmunol.1801010 30617115 PMC6327976

[15] Sloan SE, Szretter KJ, Sundaresh B, Narayan KM, Smith PF, Skurnik D, Bedard S, Trevejo JM, Oldach D, Shriver Z. 2020. Clinical and virological responses to a broad-spectrum human monoclonal antibody in an influenza virus challenge study. Antiviral Res 184:104763. doi:10.1016/j.antiviral.2020.104763 32151645

[16] Weinreich DM, Sivapalasingam S, Norton T, Ali S, Gao H, Bhore R, Xiao J, Hooper AT, Hamilton JD, Musser BJ, et al.. 2021. REGEN-COV antibody combination and outcomes in outpatients with Covid-19. N Engl J Med 385:e81. doi:10.1056/NEJMoa2108163 34587383 PMC8522800

[17] Gupta A, Gonzalez-Rojas Y, Juarez E, Crespo Casal M, Moya J, Falci DR, Sarkis E, Solis J, Zheng H, Scott N, Cathcart AL, Hebner CM, Sager J, Mogalian E, Tipple C, Peppercorn A, Alexander E, Pang PS, Free A, Brinson C, Aldinger M, Shapiro AE, COMET-ICE Investigators. 2021. Early treatment for Covid-19 with SARS-CoV-2 neutralizing antibody sotrovimab. N Engl J Med 385:1941–1950. doi:10.1056/NEJMoa2107934 34706189

[18] Lundgren JD, Grund B, Barkauskas CE, Holland TL, Gottlieb RL, Sandkovsky U, Brown SM, Knowlton KU, Self WH, et al.. 2021. A neutralizing monoclonal antibody for hospitalized patients with Covid-19. N Engl J Med 384:905–914. doi:10.1056/nejmoa2033130 33356051 PMC7781100

[19] Kallewaard NL, Corti D, Collins PJ, Neu U, McAuliffe JM, Benjamin E, Wachter-Rosati L, Palmer-Hill FJ, Yuan AQ, Walker PA, et al.. 2016. Structure and function analysis of an antibody recognizing all influenza A subtypes. Cell 166:596–608. doi:10.1016/j.cell.2016.05.073 27453466 PMC4967455

[20] Beal SL. 2001. Ways to fit a PK model with some data below the quantification limit. J Pharmacokinet Pharmacodyn 28:481–504. doi:10.1023/a:1012299115260 11768292

[21] Han A, Czajkowski L, Rosas LA, Cervantes-Medina A, Xiao Y, Gouzoulis M, Lumbard K, Hunsberger S, Reed S, Athota R, Baus HA, Lwin A, Sadoff J, Taubenberger JK, Memoli MJ. 2021. Safety and efficacy of CR6261 in an influenza A H1N1 healthy human challenge model. Clin Infect Dis 73:e4260–e4268. doi:10.1093/cid/ciaa1725 33211860 PMC8664469

[22] Deng R, She G, Maia M, Lim JJ, Peck MC, McBride JM, Kulkarni P, Horn P, Castro A, Newton E, Tavel JA, Hanley WD. 2020. Pharmacokinetics of the monoclonal antibody MHAA4549A administered in combination with oseltamivir in patients hospitalized with severe influenza A infection. J Clin Pharmacol 60:1509–1518. doi:10.1002/jcph.1652 32621543 PMC7586956

[23] Chigutsa E, Jordie E, Riggs M, Nirula A, Elmokadem A, Knab T, Chien JY. 2022. A quantitative modeling and simulation framework to support candidate and dose selection of anti-SARS-CoV-2 monoclonal antibodies to advance bamlanivimab into a first-in-human clinical trial. Clin Pharmacol Ther 111:595–604. doi:10.1002/cpt.2459 34687040 PMC8653169

[24] Magyarics Z, Leslie F, Bartko J, Rouha H, Luperchio S, Schörgenhofer C, Schwameis M, Derhaschnig U, Lagler H, Stiebellehner L, Firbas C, Weber S, Campanaro E, Jilma B, Nagy E, Stevens C. 2019. Randomized, double-blind, placebo-controlled, single-ascending-dose study of the penetration of a monoclonal antibody combination (ASN100) targeting Staphylococcus aureus cytotoxins in the lung epithelial lining fluid of healthy volunteers. Antimicrob Agents Chemother 63:e00350-19. doi:10.1128/AAC.00350-19 31138568 PMC6658777

[25] Shah DK, Betts AM. 2013. Antibody biodistribution coefficients: inferring tissue concentrations of monoclonal antibodies based on the plasma concentrations in several preclinical species and human. MAbs 5:297–305. doi:10.4161/mabs.23684 23406896 PMC3893240

[26] Momont C, Dang HV, Zatta F, Hauser K, Wang C, di Iulio J, Minola A, Czudnochowski N, De Marco A, Branch K, et al.. 2023. A pan-influenza antibody inhibiting neuraminidase via receptor mimicry. Nature 618:590–597. doi:10.1038/s41586-023-06136-y 37258672 PMC10266979

