# Supplementary material for: A phase 1 study in healthy volunteers to investigate the safety, tolerability, and pharmacokinetics of VIR-2482: a monoclonal antibody for the prevention of severe influenza A illness
Source: Antimicrob Agents Chemother. 2024 Feb 20;68(4):e01273-23. doi: 10.1128/aac.01273-23 (PMC10988998; doi:10.1128/aac.01273-23)
Supplement: Fig. S1 and S2 and Tables S1 and S2 — Nasopharyngeal PK data analyzed by alternate (M5/M7) methods. [file aac.01273-23-s0001.docx]

**Supplemental Figure 1: VIR-2482 Nasopharyngeal Pharmacokinetics Analyzed by the M5 Method**

| **Number of Results per Timepoint** | | | | | | | | | |
| --- | --- | --- | --- | --- | --- | --- | --- | --- | --- |
| **Study Days** | **1** | **2** | **3** | **8** | **15** | **29** | **57** | **85** | **239** |
| VIR-2482 60 mg | 20 | 20 | 20 | 19 | 19 | 20 | 19 | 18 | 18 |
| VIR-2482 300 mg | 20 | 19 | 20 | 20 | 20 | 19 | 19 | 20 | 17 |
| VIR-2482 1200 mg | 20 | 20 | 20 | 18 | 20 | 20 | 17 | 18 | 15 |
| VIR-2482 1800 mg | 20 | 20 | 20 | 19 | 20 | 19 | 18 | 19 | 19 |

**Supplemental Figure 1 caption:** Symbols indicate the mean and standard deviation VIR-2482 NPS concentrations at each timepoint. The number of results included at each timepoint are presented in the table below the figure.

#

# **Supplemental Table 1: Nasopharyngeal Pharmacokinetic Parameters, Analyzed by the M5 Method**

| **PK Parameter** | **VIR-2482**  **60 mg**  **(N = 20)** | **VIR-2482**  **300 mg**  **(N = 20)** | **VIR-2482**  **1200 mg**  **(N = 20)** | **VIR-2482**  **1800 mg**  **(N = 20)** |
| --- | --- | --- | --- | --- |
| C_max_ (μg/mL) |  |  |  |  |
| n | 20 | 20 | 20 | 20 |
| Geometric mean (geometric CV%) | 0.02 (1149.5) | 1.3 (96.8) | 3.8 (106.0) | 5.9 (88.5) |
| C_180_ (μg/mL) |  |  |  |  |
| n | 18 | 20 | 20 | 19 |
| Geometric mean (geometric CV%) | 0.004 (0.00) | 0.012 (4913.8) | 0.003 (105.99) | 0.248 (455.7) |
| T_max_ (day) |  |  |  |  |
| n | 20 | 20 | 20 | 20 |
| Median (min, max) | 0.9 (0.9, 28.05) | 13.4 (1.0, 83.1) | 20 (1, 56.3) | 11 (0.5, 28.2) |
| AUC_0-180_ (day*μg/mL) |  |  |  |  |
| n | 5 | 20 | 16 | 19 |
| Geometric mean (geometric CV%) | 3.62 (80.64) | 38.6 (183.0) | 199.2 (114.2) | 288 (76.6) |
| T_1/2_ (day) |  |  |  |  |
| n | 1 | 13 | 15 | 18 |
| Median (min, max) | 60.46 (60.46, 60.46) | 28.0 (7.7, 2174) | 27.58 (10.22, 2956) | 51.4 (20.9, 152.7) |

C_max_, maximum observed NPS concentration; C_180_, predicted NPS concentration at Day 180; T_max_, time to reach maximum concentration; AUC_0-180_, partial area under the concentration-time curve from time 0 to 180 days;T_1/2_, terminal elimination half-life. CV, coefficient of variation; n, number; NC, not calculated; NPS, nasopharyngeal secretion.

**Supplemental Figure 2: VIR-2482 Nasopharyngeal Pharmacokinetics Analyzed by the M7 Method**

| **Number of Results per Timepoint** | | | | | | | | | |
| --- | --- | --- | --- | --- | --- | --- | --- | --- | --- |
| **Study Days** | **1** | **2** | **3** | **8** | **15** | **29** | **57** | **85** | **239** |
| VIR-2482 60 mg | 20 | 20 | 20 | 19 | 19 | 20 | 19 | 18 | 18 |
| VIR-2482 300 mg | 20 | 19 | 20 | 20 | 20 | 19 | 19 | 20 | 17 |
| VIR-2482 1200 mg | 20 | 20 | 20 | 18 | 20 | 20 | 17 | 18 | 15 |
| VIR-2482 1800 mg | 20 | 20 | 20 | 19 | 20 | 19 | 18 | 19 | 19 |

**Supplemental Figure 2 caption:** Symbols indicate the mean and standard deviation VIR-2482 NPS concentrations at each timepoint. The number of results included at each timepoint are presented in the table below the figure.

# **Supplemental Table 2: Nasopharyngeal Pharmacokinetic Parameters, M7 Method**

| **PK Parameter** | **VIR-2482**  **60 mg**  **(N = 20)** | **VIR-2482**  **300 mg**  **(N = 20)** | **VIR-2482**  **1200 mg**  **(N = 20)** | **VIR-2482**  **1800 mg**  **(N = 20)** |
| --- | --- | --- | --- | --- |
| C_max_ (μg/mL) |  |  |  |  |
| n | 20 | 20 | 20 | 20 |
| Geometric mean (geometric CV%) | NC | 1.3 (96.8) | 3.8 (106.0) | 5.9 (88.5) |
| C_180_ (μg/mL) |  |  |  |  |
| n | 18 | 20 | 16 | 19 |
| Geometric mean (geometric CV%) | NC | NC | NC | 0.710 (86.3) |
| T_max_ (day) |  |  |  |  |
| n | 20 | 20 | 20 | 20 |
| Median (min, max) | 0.9 (0.9, 28.1) | 13.4 (1.0, 83.1) | 20 (1, 56.3) | 11 (0.5, 28.2) |
| AUC_0-180_ (day*μg/mL) |  |  |  |  |
| n | 5 | 20 | 16 | 19 |
| Geometric mean (geometric CV%) | 4.27 (98.1) | 45.1 (177.2) | 216 (109.8) | 312 (71.8) |
| T_1/2_ (day) |  |  |  |  |
| n | 0 | 7 | 11 | 17 |
| Median (min, max) | NC | 74 (51.8, 2174) | 55 (13.7, 2956) | 80.9 (30.5, 2180) |

C_max_, maximum observed NPS concentration; C_180_, predicted NPS concentration at Day 180; T_max_, time to reach maximum concentration; AUC_0-180_, partial area under the concentration-time curve from time 0 to 180 days;T_1/2_, terminal elimination half-life. CV, coefficient of variation; n, number; NC, not calculated; NPS, nasopharyngeal secretion.
