# Supplementary material for: A phase 1 study in healthy volunteers to investigate the safety, tolerability, and pharmacokinetics of VIR-2482: a monoclonal antibody for the prevention of severe influenza A illness
Source: Antimicrob Agents Chemother. 2024 Feb 20;68(4):e01273-23. doi: 10.1128/aac.01273-23 (PMC10988998; doi:10.1128/aac.01273-23)
Supplement: Appendix S1 — Study inclusion/exclusion criteria. [file aac.01273-23-s0002.docx]

# **Appendix A – Study Inclusion and Exclusion Criteria**

***Inclusion Criteria***

Each participant must meet all the following inclusion criteria at the time of randomization to be eligible for enrollment in the study:

1. Age 18 to <65 years of age, at time of randomization.
2. Healthy male or female without acute or chronic medical condition.
3. Body mass index (BMI) 18.0 kg/m^2^ to 32.0 kg/m^2^, inclusive.
4. Female participants must have a negative pregnancy test or confirmation of post-menopausal status. Post-menopausal status is defined as 12 months with no menses without an alternative medical cause. Women of child-bearing potential (WOCBP) must have a negative blood pregnancy test at screening and a negative urine pregnancy test on Day –1, cannot be breastfeeding, and must be willing to use highly effective methods of contraception 14 days before study drug administration through the last scheduled follow up visit, Week 52 Visit.
5. Male participants with female partners of child-bearing potential must agree to meet one of the following contraception requirements from the time of study drug administration until the last follow-up visit: history of vasectomy or male condom. Additionally, male participants are strongly advised to inform all female partners of the recommendation to use 1 of the contraceptive options listed for WOCBP for the same duration. Male participants must also agree not to donate sperm prior to the last scheduled follow-up visit, Week 52 Visit.
6. Non-smoker.
7. Must agree to abstain from alcohol for 72 hours and caffeine for 24 hours prior to study drug dosing.

***Exclusion Criteria***

Individuals will be ineligible if he/she meets any of the following exclusion criteria:

- - - 1. Receipt of any immunoglobulin within six months prior to randomization.
      2. Prior receipt of any monoclonal antibody.
      3. History or clinical evidence of any of the following conditions considered high risk for developing influenza-related complications.
- Residents of nursing homes and other long-term care facilities
- Established diagnosis of:
- Asthma
- Neurological and neurodevelopmental conditions (such as cerebral palsy, epilepsy [seizure disorders], stroke, intellectual disability, moderate to severe developmental delay, muscular dystrophy, or spinal cord injury)
- Chronic lung disease (such as chronic obstructive pulmonary disease [COPD], emphysema, and cystic fibrosis)
- Heart disease (such as congenital heart disease, congestive heart failure and coronary artery disease)
- Blood disorders (such as sickle cell disease and Thalassemia)
- Endocrine disorders (such as diabetes and adrenal insufficiency)
- Chronic kidney disease
- Chronic liver disease
- Metabolic disorders (such as inherited metabolic disorders and mitochondrial disorders)
- Weakened immune system due to disease or medication (such as individuals with HIV or AIDS, or cancer, or those on chronic steroids)
- People younger than 19 years of age who are receiving long-term aspirin therapy
  - - 1. History of ILI or confirmed influenza infection within three months prior to randomization.
      2. Febrile illness with or without respiratory symptoms (e.g., cough, nasal congestion) within five days prior to randomization.
      3. History of malignancy within five years (treated squamous or non-invasive basal cell skin cancers are permitted) or individual is under evaluation for malignancy.
      4. Any condition or receipt of any medication contraindicating IM injection, as judged by the investigator.
      5. History or clinical evidence of alcohol or drug abuse, within the 12 months before screening or a positive drug screen at screening unless it can be explained by a prescribed medication; the diagnosis and prescription must be approved by the investigator.
      6. History of a severe allergic reaction with generalized urticaria, angioedema or anaphylaxis within the two years prior to randomization.
      7. Person has the following laboratory parameters at screening:
      - ALT, AST, direct bilirubin, or GGT >1.2x the upper limit of normal (ULN)
      - Creatinine clearance (CLcr) <80 mL/min as estimated by Cockcroft-Gault formula
      - Hemoglobin (Hgb) <12 g/dL for males and <11 g/dL for females
- White blood cells (WBC) above the ULN or below the lower limit of normal (LLN) (Note: Individuals with benign ethnic neutropenia may be enrolled)
  - - - Platelets below the LLN
- Internal Normalized Ratio (INR), prothrombin time (PT) or partial thromboplastin time (PTT) above the ULN
  - - 1. People in whom nasopharyngeal swabbing is not tolerated or possible, or history of frequent epistaxis (nose bleeds).
      2. Received an investigational agent within 90 days before study drug administration or are currently participating in another interventional study. People taking part in any other study at any time during participation in this study, inclusive of the follow-up period.
      3. Use of prescription or over-the-counter (OTC) drugs or herbal supplements in the 14-day period prior to Study Day 1 (except for Paracetamol (acetaminophen) ≤2 g/day, aspirin ≤3 g/day or ibuprofen <1.2 g/day, and hormonal birth control by female participants).
      4. Laboratory evidence of active infection with hepatitis B virus (HBV), hepatitis C virus (HCV) or human immunodeficiency virus (HIV) at the time of study screening.
